# Experimental Evidence of Threat-Sensitive Collective Avoidance Responses in a Large Wild-Caught Herring School

**DOI:** 10.1371/journal.pone.0086726

**Published:** 2014-01-29

**Authors:** Guillaume Rieucau, Kevin M. Boswell, Alex De Robertis, Gavin J. Macaulay, Nils Olav Handegard

**Affiliations:** 1 Institute of Marine Research, Bergen, Norway; 2 Florida International University, Biscayne Bay Campus, Marine Sciences Building, North Miami, Florida, United States of America; 3 National Marine Fisheries Service, Alaska Fisheries Science Center, National Oceanic and Atmospheric Administration, Seattle, Washington, United States of America; University of Plymouth, United Kingdom

## Abstract

Aggregation is commonly thought to improve animals' security. Within aquatic ecosystems, group-living prey can learn about immediate threats using cues perceived directly from predators, or from collective behaviours, for example, by reacting to the escape behaviours of companions. Combining cues from different modalities may improve the accuracy of prey antipredatory decisions. In this study, we explored the sensory modalities that mediate collective antipredatory responses of herring (*Clupea harengus*) when in a large school (approximately 60 000 individuals). By conducting a simulated predator encounter experiment in a semi-controlled environment (a sea cage), we tested the hypothesis that the collective responses of herring are threat-sensitive. We investigated whether cues from potential threats obtained visually or from the perception of water displacement, used independently or in an additive way, affected the strength of the collective avoidance reactions. We modified the sensory nature of the simulated threat by exposing the herring to 4 predator models differing in shape and transparency. The collective vertical avoidance response was observed and quantified using active acoustics. The combination of sensory cues elicited the strongest avoidance reactions, suggesting that collective antipredator responses in herring are mediated by the sensory modalities involved during threat detection in an additive fashion. Thus, this study provides evidence for magnitude-graded threat responses in a large school of wild-caught herring which is consistent with the “threat-sensitive hypothesis”.

## Introduction

Schooling behaviour has been considered primarily as an adaptation that confers security advantages to gregarious fishes [1]–[3] through the action of several mechanisms such as a greater power of predator detection [4], [5], the numerical dilution of risk and abatement effect [1], predator confusion [6], [7] or coordinated evasive manoeuvres [1], [8]. However, making adaptive antipredator decisions in spatially and heterogeneous environments requires that prey collect and act upon accurate information. Schooling has been suggested to allow rapid evasive reactions and significant effort has been directed at ascertaining the mechanisms that underlie efficient predator detection in schooling fish [3], [5], [9]. Schooling fish can learn about immediate threats using visual [10], [11], chemical [12], [13] acoustical or hydraulic cues, sensed by the mechanoreceptors located in the lateral line system, that emanate from the predator's swimming movement [14], [15] or indirectly from the sensory information produced by the avoidance behaviour of risk-aware school members [16], [17]. Several studies have investigated how predator information can spread across a whole school [18] in such a way that the information transfer outpaces the speed of an approaching predator [9], [18] or the swimming speed of any fish within the school ensuring a rapid propagation of predator cues. A rapid transfer of threat information across a fish aggregation regardless of the size of the aggregation is a fundamental component of safety-enhancement for schooling fish. Several mechanisms have been proposed to explain an efficient transmission of undamped information within schools: waves of agitation [19]–[21], fast pressure pulses emitted by startled fish [22], and also compressional density waves that can occur over very large distances, e.g. 10's to 100's of km [21], [23].

In aquatic ecosystems, prey can detect the presence, location, and the nature of a threat from cues obtained through diverse sensory channels acting independently or in an additive manner [24], [25]. Under natural conditions, animals may simultaneously receive multiples sources of sensory information from a potential threat. Typically, it is assumed that combining cues from multiple sensory modalities enhances the accuracy of an animal's decisions [26]. In particular, animals can combine inputs from several sensory cues in order to assess risk, thus minimizing the cost of making erroneous antipredator decisions. Therefore, an animal able to access multiple cues from a potential threat should improve its assessment of local risk leading to more accurate antipredatory responses: the well-documented “sensory complement” hypothesis [24], [27]–[30] suggests that the integration of multiples sensory inputs can interact in an additive or synergistic way. Prey often exhibit threat-sensitive antipredatory responses, with the strength of antipredatory responses proportional to the magnitude of the sensory inputs received in one or several pathways expressed in one or several sensory modalities [29], [31]–[34]. For example, in an earlier experiment, Helfman [33] reported that damselfish (*Stegastes planifrons*) tune their antipredator responses to the magnitude of the predatory threat.

Despite the growing body of published results giving support to the “threat-sensitive” hypothesis, most of these studies have been conducted either on solitary prey or small prey groups. For instance, Magurran and Pitcher [35] showed that when in shoals (up to 50 individuals), minnows (*Phoxinus phoxinus*) modified their predator-evasion behaviours when under escalating attack by a predatory pike (*Esox lucius*). In addition, by testing juvenile convict cichlids (*Archocentrus nigrofasciatus*) in different group sizes (1, 3 and 6 fish), Brown et al. [36] found that group size influences the strength of threat-sensitive responses in a way that only the larger groups (6 fish) exhibited graded predator-evasion responses consistent with the threat-sensitive hypothesis. Even though the effect of increasing group size on graded avoidance behaviours is well established for small aggregations, it remains unknown whether this is applicable to larger aggregations. It is unclear whether massive aggregations, such as schools of pelagic fishes that can reach shoal sizes up to several million individuals [37], [38], display threat-sensitive responses. Knowledge of the sensory mechanisms that mediate collective antipredatory responses in large schools is very limited mostly due to the difficult task of observing and quantifying large-scale dynamic behavioural patterns of fish prey and the interaction with their predators in natural conditions.

In this study, we explored the sensory modalities that mediate collective antipredator responses of wild-caught Norwegian Spring Spawning (NSS) herring (*Clupea harengus*) when in a large shoal (approximately 60 000 individuals). We tested the hypothesis that herring collective responses are threat-sensitive by conducting a simulated predator encounter experiment in a semi-controlled environment (sea cage). We investigated whether cues obtained via different sensory pathways (vision or perception of water displacement) affected the strength of collective avoidance reactions. A common antipredator strategy employed by schooling herring in the wild is to dive when threatened [39]–[41]. We observed and quantified the diving behaviour of herring using acoustics, which is well-suited to quantify this behaviour [42]. We manipulated the sensory nature of the simulated threat by presenting 4 predator models that differed in shape and colour with the assumptions that black-coloured models (V+) would be more conspicuous against the visual background than transparent models (V−) and that predator-shaped models would produce a larger bow-wave (BW+) that can be detected by fish compared to flattened models which would induce less water displacement when in motion underwater (BW−). Stronger collective avoidance responses were expected when both sensory cues were combined compared to when weaker sensory cues were present.

## Material and Methods

### NSS herring school and housing facilities

**Figure 1:**
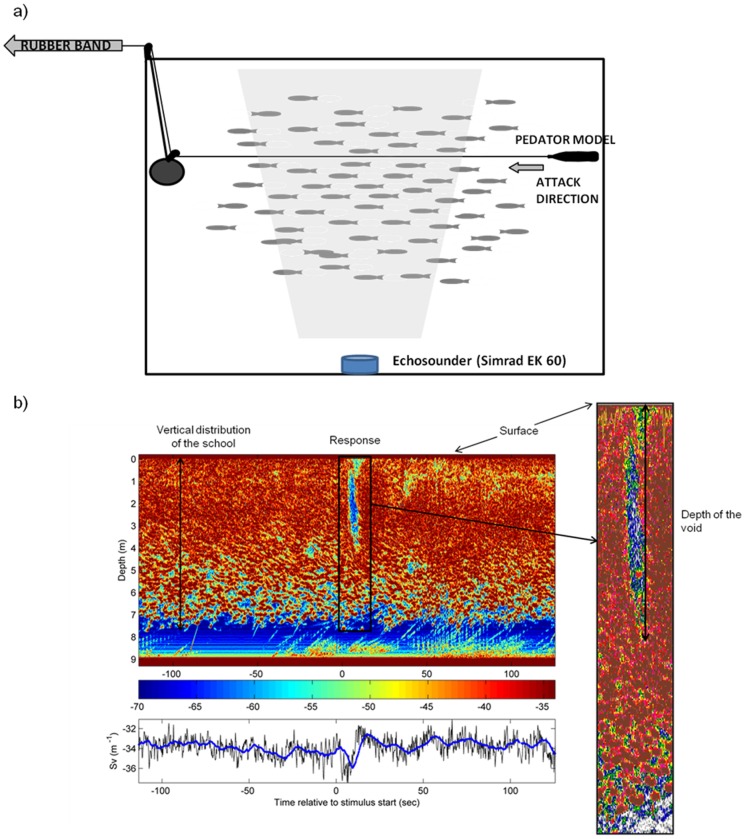
Apparatus located in the experimental net pen. Attacks were simulated using 4 different models. An elastic cord was attached to the models and the release of tension of the cord induced the motion of the models through the herring school at 1(a). We measured the strength of the collective response using an upward-looking 120 kHz split-beam echosounder (Simrad EK 60) placed at the bottom of the pen. (b) Example of the quantification procedure of the vertical collective response strength from an echogram. Also presented vis a time series of the volume backscattering coefficient (Sv), expressed in dB re 1 m^−1^ [44], relative to the start of the stimulus.

In April 2012, 14 tonnes of adult NSS herring were caught by a commercial purse-seine fishery vessel as part of the vessel's fishing quota, on the west coast of Norway in 2 separate fishing operations (8 tonnes in Bårsundet 60°00′05 N 05°29′20 E and 6 tonnes in Søredvågen 59°57′40 N 05°29′57 E) and towed using a specially designed towing pen to the Institute of Marine Research aquaculture facility located in Austevoll, Norway. The school was placed in a rectangular sea cage (net pen: 12 m long×12 m wide×12 m deep, figure 1a) at the end of a dock located in a North Sea fjord (60°5′20 N 05°15′58 E). The fish were held in the net pen for 3 months prior to the experiments to allow them to acclimate. They were fed with standard small-sized aquaculture pellets in addition to any naturally available prey that flowed into the pen. Prior to the experiment, we measured the body length and weight of 155 herring caught from the housing sea cage using a landing net (N = 155; body length = 31.39±2.17 cm; weight = 219.25±50.21 g; index of fish condition (100×weight×body length^−3^) = 0.702±0.09; all results are expressed as a mean ± SD) (similar to [43]). The experiment described in this paper was conducted between July 9 and July 10 2012.

The Institute of Marine Research is permitted to conduct experiments at the Austevoll aquaculture facility by the Norwegian Biological Resource Committee (Biologisk Ressurskomite, BRK) and the Norwegian Animal Research Committee (Forsøksdyrutvalget). NSS herring is not an endangered or protected species and the Norwegian Directory of Fisheries allocated the fishing quota used to capture the fish and permitted the holding of herring in net pens at the Austevoll aquaculture facility. In accordance with the Norwegian Animal Research Committee's regulations, our study did not require a specific prior allowance as no pain or discomfort was caused to the captive animals. However, our study was approved by the person in charge of fish welfare (Sjur Åge Skår) at the Austevoll aquaculture facility who evaluated our experiment as ethically acceptable.

### Apparatus

We used 4 different predator models to simulate attacks of a solitary predator: 1) a black-coloured predator-shaped model (V+/BW+); 2) a transparent predator-shaped model (V−/BW+); 3) a black-coloured flat model (V+/BW−) and finally; 4) a transparent flat model (V−/BW−). We built the 2 predator-shaped models from plastic bottles (35 cm long×9 cm wide). One bottle was covered with water resistant black duct tape while the second bottle was kept transparent. Two other models (35 cm long×9 cm wide×1.5 cm thickness) were built from transparent Plexiglas in a way that approximated the shape of the bottles when flattened. One model was also covered with black duct tape while the second flat model was kept transparent. We assumed that the black-coloured models would provide more visual contrast and be more visually conspicuous than the transparent ones, and that the two flat models differed from the predator-shaped models by creating less water displacement when in motion underwater.

**Figure 2:**
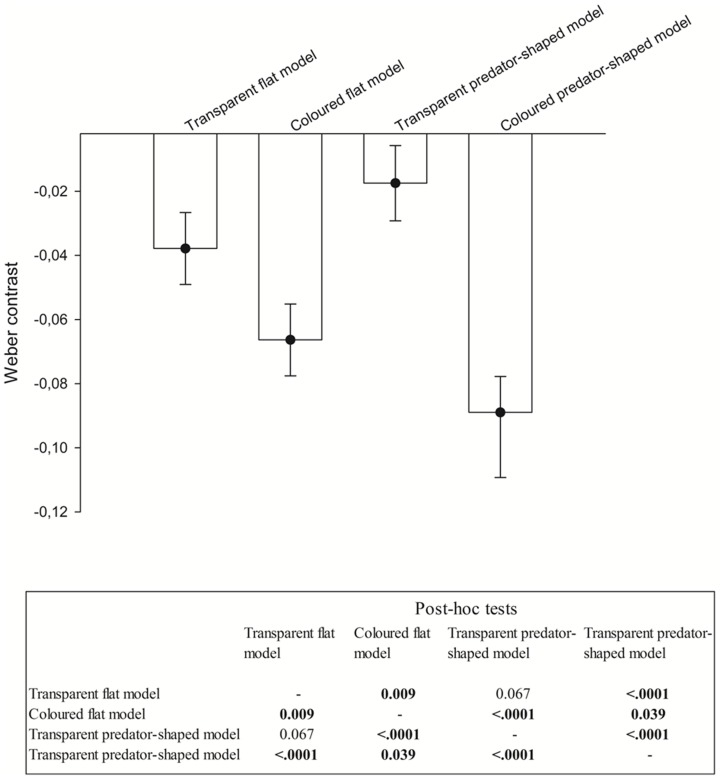
Visibility assessment of the 4 models. Four fixed images, in which the models were distinguishable from the background, were converted to grayscale intensities (I) using Matlab (Mathworks). We calculated the Weber contrast ((I_model_ – I_background_)/I_background_) as a metric where I_model_ and I_background_ are the mean pixel intensities within the model outline and of adjacent pixels where no fish are present, respectively. Transparency was then used as a measure of visual conspicuousness of the 4 models. Table shows the results of Tukey's *post hoc* analyses to identify differences in Weber contrast between models. Significant differences in Weber contrast are presented in bold.

To quantify the transparency of the predator models an upward-looking high resolution colour LED underwater camera located at 10 m depth was used (image sensor Sony Super HAD CCD, PAL: 752 (H)×582 (V), lens monofocal fixed iris 4.3 mm, shutter speed 1/50∼1/100000 sec, 500 TV lines). For each model, we extracted 4 fixed images in which the model was distinguishable from the background and converted them to grayscale intensities (I) using Matlab (Mathworks). The Weber contrast, defined as (I_model_ – I_background_)/I_background_, was used to quantify visibility, where I_model_ and I_background_ are the mean pixel intensities within the model outline and of adjacent pixels where no fish are present, respectively. When presented to the school, the models are not running in a straight line leading to visual stimulation for the fish in front as well as below the model, and we use the transparency as a measure of visual conspicuousness. We tested whether the 4 models differed in conspicuousness using a one-way ANOVA (F_3,12_ = 37.35, *p*<.0001; figure 2). In addition, we used Tukey's *post hoc* analyses to identify differences in Weber contrast between models (figure 2). No significant difference in Weber contrast was detected between the two transparent models (Tukey's *post hoc p* = 0.067). Moreover, Weber contrasts for these two models were lower than those of the two black-coloured models suggesting that the transparent flat and the transparent predator-shaped models were less conspicuous than the 2 black-coloured other models, which were more visible against the visual background.

The flat model represented a different visual stimulus than the predator-shaped model. If we assume a uniform yaw angle (assuming a rectangle, i.e. disregarding the pointy front end), the mean projected area side aspect projection is expressed as:
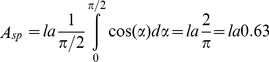
, where l is the length of the model (35 cm) and a is the diameter/width (9 cm). This suggests than when viewed from the side, the projected area of the flat model is ∼64% of that of the predator-shaped model. A similar approximation can be used for the front aspect. The path of the model was not perfectly straight, and from observations obtained from an imaging sonar (DIDSON, Soundmetrics, Corp.) recording horizontally on the path of the different models, the typical off path angle was ±15° degrees. By calculating the maximum forward projected area as 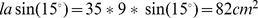 and assuming a sinusoidal motion, we computed the mean projected area by integrating and normalizing over half a period:
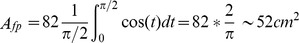
For comparison, the area of the predator-shaped model (without the sinusoidal motion) was 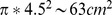. In combination, a conservative estimate is that the flattened model had a projected area of 60% of that of the predator-shaped model. Therefore, the black-coloured flat model presented, on average, at least less 60% projected area than the predator-shaped model, and the transparent predator-shaped model had only 19% of the contrast of the black-coloured predator-shaped model.

To simulate a predator attack, the models were pulled transversally across the pen at 1 m depth by attaching fishing line to the model and leading it through a block at the arriving end of the pen (figure 1a). The fishing line was attached to an elastic shock cord which was extended to constant length and attached to a fixed point located 20 m from the net pen. The models were moved across the school by releasing the shock cord, which moved the model through the top of the school across the net pen. Because of their drag differences, the speed of the flat models was matched up to the speed of the predator-shaped models by manually towing the model along the same path by walking the fishing line along the dock. The speeds were recorded by timing the tow duration (approximately 3.40 m/s) and were consistent between trials.

We measured the magnitude of the water disturbance caused by the models in a separate experiment that duplicated the net pen setup, but on a smaller scale in a tank of water (3 m long×1.8 m wide×1.2 m deep). The velocity of water displacement caused by the motion of two models (V+/BW+ and V+/BW−) was measured using an Acoustic Doppler Velocimeter (ADV. Nortek AS, Rud, Norway), sampling at 25 Hz. Measurements of velocity were taken at a point 177 mm above the surface of the model as it was pulled past the fixed sensor at a speed of 3.40 m/s and repeated 10 times for each predator model. Control measurements were also taken of the apparatus without any model (i.e., fishing line only). The mean of the maximum observed water velocity for each run was calculated and used as an indication of the relative water disturbance caused by the two model shapes. The V+/BW+ model generated an outward flow of water with a mean maximum magnitude of 0.073±0.018 m/s (mean ± SD) while the V+/BW− model generated a flow of 0.033±0.017 m/s. The velocities from control runs were indistinguishable from the background noise level (mean magnitude of 0.024 m/s).

To measure the strength of the collective diving response, we mounted an upward-looking 120 kHz split-beam echosounder with a 7 degree beamwidth (Simrad EK 60, Kongsberg Maritime AS, Horten, Norway) on a gimbal close to the bottom of the pen. The data were imported into Echoview 5.2 software (SonarData Pty. Ltd., Tasmania, Australia), and the extent of the diving behaviour was estimated by manually identifying the depth of the perturbation in the echogram (measuring the vertical dimension of the void: figure 1b). In cases where no perturbation was identified, the response variable was classified as “no reaction”. The response was then separated into the probability of response, combined with, in the case of a response, the strength of that response.

The complete experiment consisted of 5 series of measurements with each series consisting of measurements of the 4 models and a control treatment (i.e. the fishing line walked down the dock without a model), for a total of 25 tests. The control treatment allowed testing whether the noise from the releasing gear, activity on the dock, and the motion of the fishing line may have caused fish avoidance responses. During each experimental series, herring were exposed once to each experimental treatment in random order. Based on initial observations, we established a 6 minute interval between successive treatments in the same experimental series, which was sufficient for the fish to school in a similar fashion as prior to exposure. We used a paired t-test to compare an estimate of school density (sv: volume backscattering coefficient expressed in m^−1^ [44]) before (1 minute) and after (3 minutes) the exposure to the experimental treatments. We calculated sv values from **Sv** estimates (i.e. volume backscattering strength in logarithmic domain expressed in dB re 1 m^−1^) obtained from echograms in Echoview using the following relationship: **Sv** = log_10_ (sv), thus sv = 10^(**Sv**/10)^ [44]. No significant differences in sv before and after exposure was detected (n = 25, t = −1.017, df = 24, *p* = 0.319) suggesting that, after 3 minutes, the school went back to a similar swimming dynamic and internal structure than before being exposed to the different models. The volume backscatter from our experimental herring school (**Sv** = −33.19 dB re 1 m^1^) was equivalent to that observed of 2 wild herring schools in the Norwegian Sea outside the reproduction and feeding periods (wild school I: **Sv** = −36.5 dB re 1 m^−1^; wild school II **Sv** = −33.2 dB re 1 m^−1^ [45]), which indicates that the penned herring were under realistic packing densities. We conducted 3 experimental series on the first day of the experiment (15 tests) and 2 experimental series on the second day (10 tests) with consecutive experimental series separated by at least 5 hours.

A primary objective of our study was to investigate the collective response to multi-sensorial stimuli in herring group sizes that match the social conditions in the open ocean. This posed logistical restrictions as it is was not feasible to create smaller subsets to control for pseudoreplication [46], as is common practice in smaller scale experiments. However, due to the large number of herring in our experimental net pen and their highly dynamic swimming pattern, it is likely that this has created a substantial mixing of individuals ensuring that different individuals directly encountered the predator models or the fishing line in each trial.

### Statistical analysis

We tested if the vertical distribution of herring prior to exposure changed among the experiments using a one-way ANOVA. We conducted a two-way ANOVA to investigate the effect of the colour (two levels: black-coloured or transparent) and shape (2 levels: flat or bottle shaped) on the strength of the collective responses, as well as the interaction between these two factors. We used Tukey's *post hoc* analyses to identify differences between treatments. All analyses were conducted with Statistica 11 (StatSoft, Inc. Tulsa, Oklahoma, USA). Hereafter, all results are expressed as a mean and its standard error.

## Results

**Figure 3:**
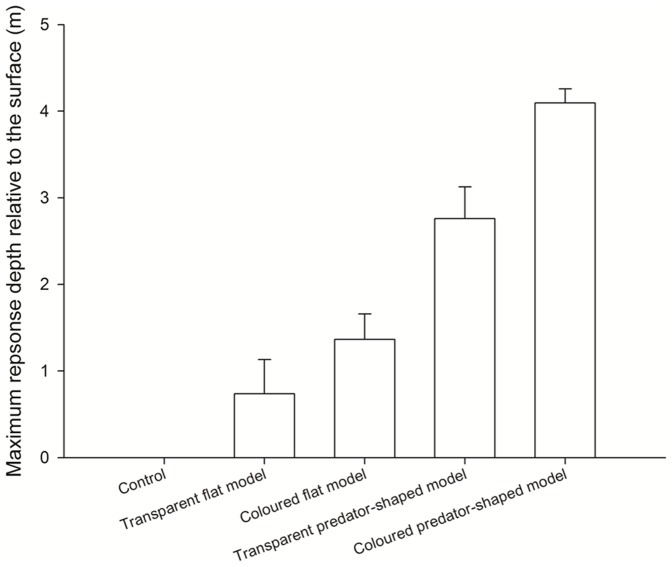
Maximum depth of the collective responses (mean ± SEM) to the different predator models showing magnitude-graded threats responses. Stronger vertical avoidance responses were observed when the herring school was exposed to the black-coloured predator-shaped model, which is more visible and produces a stronger hydrodynamic stimulus, compare to when the herring school exposed to the flat and/or transparent models and to the control.

The vertical distribution of fish before in the net pen prior to each treatment did not vary by treatment type (F_4,20_ = 0.13, *p* = 0.96; table 1) suggesting that initial conditions were similar across treatments. The probability of a collective avoidance response differed significantly among the treatments (F_4,20_ = 16, *p*<.0001; table 1). There was a significant effect of both model shape (F_1,16_ = 55.72, *p*<.0001) and colour (F_1,16_ = 16, *p* = .007) on the vertical extent of the diving response. No significant interaction was detected between model shape and colour (F_1,16_ = 1.25, *p* = .279). Stronger avoidance reactions were observed when the school was exposed to the black-coloured predator-shaped model compared to the other 3 models and the control (Tukey's *post hoc* tests: V+/BW+>V−/BW+, *p* = 0.025; V+/BW+>V+/BW−, *p*<.0001; V+/BW+>V−/BW−, *p*<.0001; V+/BW+>control, *p*<.0001; figure 3). Stronger avoidance responses were observed when the school was exposed to the transparent predator-shaped model compared to the two flat models and the control (Tukey's *post hoc* tests: V−/BW+ vs. V+/BW−, *p* = 0.019; vs. V−/BW−, *p* = 0.001; vs. control, *p*<.0001; figure 3). No statistically significant difference was found between the response strength induced by the two flat models (Tukey's *post hoc* tests: V+/BW+ vs. V−/BW− *p* = 0.51; figure 3). The control treatment never induced avoidance reactions.

**Table 1:** Mean school vertical distribution prior to stimuli exposure and collective response strength to the 4 predator models.

		N	Response probability	School vertical distribution before exposure (m)		Collective response depth (m)	
Model	Stimuli			Mean	Standard error	Mean	Standard error
Control	fishing line	5	0	7.24	0.174	Absence of response	
Transparent flat model	V−/BW−	5	0.6	7.28	0.122	0.74	0.395
Coloured flat model	V+/BW−	5	1	7.31	0.115	1.36	0.295
Transparent predator-shaped model	V−/BW+	5	1	7.15	0.221	2.76	0.368
Coloured predator-shaped model	V+/BW+	5	1	7.18	0.223	4.09	0.162

The 4 predator models consisted of a black-coloured predator-shaped model (V+/BW+); a transparent predator-shaped model (V−/BW+); a black-coloured flat model (V+/BW−) and a transparent flat model (V−/BW−).

## Discussion

Herring exhibited increasing avoidance responses to more visible models that produced a stronger hydrodynamic stimulus compared to the flat and/or transparent models. This suggests that the combination of cues from different sensory modalities elicited the strongest avoidance reactions. Thus, our results show that herring collective antipredator responses are mediated by the sensory modalities involved during threat detection. This study provides empirical evidence for magnitude-graded threat responses in a large school of herring consistent with the threat-sensitive hypothesis [33].

Stronger diving responses were observed to the predator-shaped models than the flattened models. Although the predator-shaped models generated more water flow and a larger hydrodynamic signal, they also presented a larger visual target as the flattened models presented a ∼60% lower projected area than the predator-shaped models. Given that multiple factors were altered simultaneously in this case, it is difficult to determine how much of the increased response to the predator-shaped model compared to the flat models is attributable to the larger hydrodynamic signal and how much is attributable to increased visibility. Given that the black-coloured predator-shaped model elicited a larger response than the lower-contrast transparent predator-shaped model, one can deduce that the Weber contrast plays a significant role in the response. When comparing the flat coloured model to the transparent predator-shaped model, the predator-shaped model produced a much larger hydrodynamic signal than the flat model, but it is unclear if the predator model was also more visually conspicuous as the transparent predator-shaped model had a larger projected area but a significantly lower Weber contrast. Thus, although it cannot be determined precisely, it is likely that the increased response to the predator models relative to the flat models is attributable to both the elevated hydrodynamic and possibly the increased visibility of the predator model.

In the case of reduced hydrodynamic cues produced by the flat models, avoidance responses did not depend on model colouration, which may suggest that information obtained visually was insufficient alone to elicit strong collective avoidance responses in the schooling herring. Vision is commonly considered as one of the most efficient modalities for the initial localisation of predators in aquatic environments [10], [34], [47], as well as an important sensory mechanism promoting schooling behaviour [48]. However, the results of our study suggest that visual cues must be combined with another sensory modality to increase the strength of the collective responses. In aquatic habitats, environmental factors, such as water turbidity, can strongly affect visually mediated predator-prey interactions. Water turbidity was found to alter transmission of visual information in fathead minnows (*Pimephales promelas*) in such a way that some fishes were unable to observe the antipredator behaviours of school mates, increasing then their vulnerability to an attack [49]. It is worth noticing that our study was conducted during summer where low underwater visibility may have reduced the efficiency of visual detection. Strong seasonal patterns in herring antipredator behaviours have been previously reported [42], [50], [51] with lower responsiveness to audio-playbacks of predator calls reported after spawning and during feeding periods (late spring and summer) compared to winter [52], [53]. Consequently, seasonal variations in the general tradeoffs between survival, feeding and reproduction experienced by herring coupled with changes in environmental factors can affect the sensory mechanisms at work during predator-prey interactions.

In low visibility conditions, other sensory modalities such as cues detected by the olfactory system [13], [24], [25], [27], [30], the lateral line, and the auditory system are likely to play an important role during predator encounters [54]. Even though we observed stronger collective responses from models which produced stronger stimuli in multiple sensory modalities, only a fraction of the school exhibited avoidance responses. A possible explanation may be that one or several sensory modalities that fish may rely on during their assessment of risk are weak or absent from the predatory models. In our study, we only focused on modalities that spread rapidly in the environment such as visual and hydrodynamic cues [55]. However, we did not consider chemical cues that spread more slowly, persist over a longer period of time in the environment and that fish prey also use to locate predators [56], [57] and which can act independently or in synergy with other modalities as vision [24], [25], [27], [30].

Recent research in collective behaviour provides a mechanistic explanation for how information can spread among an entire animal aggregation no matter how large the aggregation is. An expected property of animal groups is that the flow of collective information is unconstrained by the group size but may be achieved by scale-free behavioural correlations [58], [59]. This has been recently used to explain large scale coordinated antipredator events observed in European starlings flocks (*Sturnus vulgaris*) [58] and Gulf menhaden schools (*Brevoortia patronus*) [60]. The collective behaviour framework gives an explanation of how a group reacts as a whole to environmental perturbations and accounts for the undamped long-range transfer of information among all group members, even in very large animal aggregations.

While our study revealed graded vertical avoidance reactions, only a fraction of the school reacted and exhibited avoidance responses regardless the nature of the threat stimuli presented (see table 1); a result that contrasts with the scale-free collective responses [58], [61]. Considering the high density of fish in the net pen, it is likely that as the distance from the model increased, the direct detection of the model by individual fish decreased, and that, responses far from the predator model are most likely induced by neighbouring fish's reaction [18]. Consequently, the observed differences in collective responses to the models' characteristics may reflect differences in information transfer strength. A plausible explanation is that the extent to which information is conveyed through the school depends on the initial number of individual fish that responded to the experimental stimuli (number of early responders [18]), which may determine the strength of the collective reactions. It has been shown that the strength or the characteristics of a stimulus (e.g. orientation or distance [16]) can affect the perception of the threat level and hence the strength of the induced responses [62], [63]. Yet, to date, we cannot clearly ascertain whether the responses observed were triggered by the direct stimuli presented or by the evasive reactions of neighbouring fish. More efforts towards a finer investigation of the avoidance responses with a particular focus on the timing of the individual's response latency (short-latency vs. long latency responses similar to [16]–[18]) are warranted to ascertain the functional role of information transfer in schooling fish.

The behavioural antipredatory responses of aggregated fish can affect the outcome of predator-prey interactions with important subsequent effects on both prey populations and communities. Acoustics provides an efficient means to address some of these questions by precisely observing and quantifying large-scale collective antipredator behaviours in schooling fishes and the interaction with their predators [60]. By applying these observation techniques in a controlled setting, we found evidence for sensory complementation and threat-graded avoidance reactions in a large school of marine fish, increasing our knowledge of the mechanisms underlying collective evasive responses in large animal aggregations.
